# Gravitational cues modulate the shape of defensive peripersonal space

**DOI:** 10.1016/j.cub.2016.09.025

**Published:** 2016-11-07

**Authors:** Rory John Bufacchi, Gian Domenico Iannetti

**Affiliations:** 1 Department of Neuroscience, Physiology and Pharmacology, University College London, London, WC1E 6BT, UK; 2 Centre for Mathematics and Physics in the Life Sciences and EXperimental biology (CoMPLEX), University College London, London, WC1E 6BT, UK

## Abstract

The potential damage caused by an environmental threat increases with proximity to the body, so animals perform more effective and stronger defensive responses when threatening stimuli occur nearby the body, in a region termed the defensive peripersonal space (DPPS) 1, 2. We recently characterized the fine-grained geometry of the face’s DPPS by recording the enhancement of the blink reflex elicited by electrical stimulation of the median nerve (hand-blink reflex, HBR), when the hand is closer to the face [3]. The resulting DPPS has the shape of a bubble, elongated asymmetrically along the rostro-caudal axis, extending further above eye-level [4]. We hypothesized that this vertical asymmetry is determined by gravitational cues: the probability that a threat will hit the body is higher when it comes from above. By systematically altering body posture, we show that the extent of DPPS asymmetry is defined in an earth-centred coordinate frame. This observation suggests the brain takes gravitational cues to automatically update threat value in an adaptive mechanism that accounts for the simple fact that objects fall down.

## Teaser

Bufacchi and Iannetti show that gravitational cues affect the shape of the defensive peripersonal space. They come to this conclusion by measuring how the magnitude of the eye-defensive blink reflex changes as a function of the position of external threats and participants’ body posture.

## Main Text

In Experiments 1 and 2, participants were upright (Figure S1 in the Supplemental Information). We electrically stimulated the right wrist, while the right hand was placed in a total of 10 positions on a coronal plane located 4 cm from the nose. In Experiment 1 these positions were along the body midline: ‘far-low’, ‘low’, ‘middle’, ‘high’ and ‘far-high’, symmetrically with respect to eye-level (Figure S1). In Experiment 2 these positions were along a horizontal line at eye-level: ‘far-right’, ‘right’, ‘middle’, ‘left’ and ‘far-left’, symmetrically with respect to the midline. Because preliminary experiments indicated that effort contributes to HBR magnitude, the participants’ arm was kept in place by the experimenter, and participants were instructed to relax their arm muscles.

**Figure 1:**
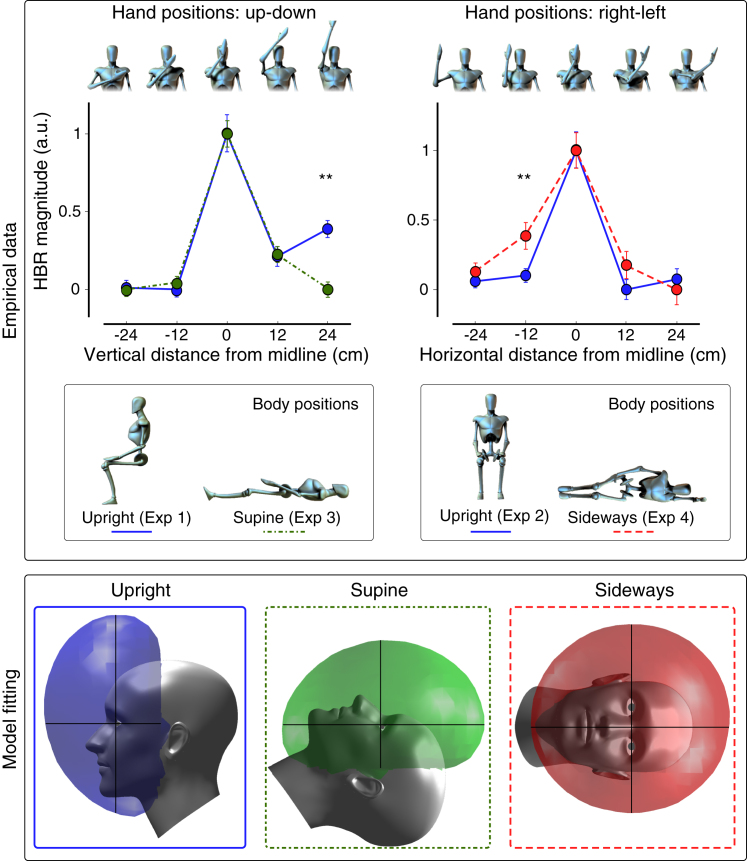
Effect of gravitational cues on DPPS shape. Top panel: HBR magnitude following stimulation of the hand in different positions (top row of figurines; see also Figure S1). HBR magnitude is expressed as Z-scores within-subject, and normalised between 0 and 1 within-experiment. HBR magnitude was overall larger when the stimulated hand was above the head in earth-centred coordinates, regardless of body position. Error bars indicate the standard error of the mean (SEM). Asterisks indicate: ^∗^p < 0.05, ^∗∗^p < 0.01, ^∗∗∗^p < 0.001. All post-hoc statistical comparisons are reported in Supplemental Figure S1 and Table S1. While the largest HBR magnitude was always observed when the hand was in position ‘middle’ (0 cm), body posture clearly modulates the HBR magnitude, and therefore alters the shape of the DPPS on the basis of gravitational cues. Bottom panel: HBR magnitudes were used to derive a fine-grained map of DPPS through a formal geometrical model fitting to the HBR data [4]. The three different bubbles represent the DPPS shape as iso-threat surfaces. They define the set of hand positions at which the modelled HBR magnitude is the median between the minimum and maximum measured magnitudes. Note that the bubble always extends upwards in earth-centred coordinates — against the direction of gravity, regardless of body position. These findings indicate that the nervous system continuously updates the threat value of environmental stimuli, taking into account gravitational cues, and thus automatically inferring the effects of physical laws of nature.

If the hypothesis that the DPPS vertical asymmetry is determined by gravitational cues is correct, the shape of the DPPS should remain asymmetric along the gravity axis, regardless of head orientation. Alternatively, the DPPS could remain asymmetric along the head vertical axis, regardless of head orientation with respect to gravity. To distinguish between these two possibilities, we altered body posture relative to the direction of gravity, and derived the geometry of the DPPS. In Experiment 3, participants lay supine. In Experiment 4, participants lay on their left side (Figures 1 and S1). Hand positions in head-centered coordinates were identical to Experiments 1 and 2, respectively (for further methodological details see the Supplemental Information).

A 5 x 2 repeated-measures ANOVA on the data pooled from Experiments 1 and 3 showed significant effects of ‘hand-position’ (F = 46.293, p < 0.0001) and a significant ‘hand-position’ x ‘body-position’ interaction (F = 2.7512, p = 0.034). This interaction arose from a larger HBR magnitude in hand position ‘far-high’ (t = 3.7617, p = 0.0013) when participants were upright. So when an individual is supine, the DPPS shape is no longer elongated equally far above eye level, but becomes less asymmetrical along the head vertical axis (Figure 1).

A 5 x 2 repeated-measures ANOVA on the data pooled from Experiments 2 and 4 showed significant main effects of ‘hand-position’ (F = 32.776, p < 0.0001) and ‘body-position’ (F = 11.996, p= 0.0025). This latter main effect arose from a larger HBR magnitude in hand position ‘right’ (t = –2.929, p = 0.0083) when participants were lying on their side. Therefore, when individuals lie on their left side, although the HBR magnitude remains largest in position ‘middle’, the HBR increase in position ‘right’ indicates that the DPPS shape becomes asymmetrical: it has a larger extent on the right side — opposite to the direction of gravity (Figure 1). All statistical comparisons are detailed in the Supplemental Table and Figure.

To formally test the effect of gravity on DPPS shape, we used three versions of a geometric model of the DPPS [4], in which the HBR magnitude is dictated by the probability of the face being hit by a threat. In the ‘balloon’ version, gravitational cues influence DPPS shape: the DPPS always extends upwards like a helium-filled balloon. In the ‘helmet’ version, there is no influence of gravitational cues on DPPS shape: the DPPS moves along with the head like a helmet. Both versions were accepted (‘helmet’: p = 0.094, GoF = 1.30; ‘balloon’: p = 0.079, GoF = 1.42; see Supplemental Information for the meaning of p and GoF values). A third, alternative version postulating no DPPS asymmetry in any body posture was rejected (p = 0.020; GoF = 2.07). In other words, the DPPS behaves partially as a balloon, and partially as a helmet.

Taken together, these results clearly support the notion that the brain uses a malleable DPPS representation, and continuously updates the threat value of stimuli based on gravitational cues, automatically inferring the effects of physical laws. The ability of the nervous system to adjust the DPPS shape based on gravitational cues has a clear survival advantage. Gravity causes all objects to fall: in natural environments a threatening object is more likely to cause damage when it is above the body than when it is below — a fact obviously independent of body posture. Therefore, heightened defensive responses to above-body threats, which have greater hit probability, would maximally mitigate harm. There are a few hints of a vertical asymmetry in the threat value assigned to environmental events. Vertical asymmetries in visual perception are well documented [5] and, as an example more directly related to threatening stimuli, larger sympathetic skin responses are elicited by a visual threat approaching vertically rather than horizontally [6].

Adjustment of the HBR magnitude results from a top-down cortical modulation of the excitability of brainstem interneurons [3]. The modulation of the DPPS shape due to gravity, then, likely relies upon the dense vestibular, somatosensory and visual information received by cortical areas representing the DPPS, even at single-cell level 1, 7, which include the ventral intraparietal sulcus and F4 regions 1, 2, 8. The location of threats in such a gravity-adjusted map defines their harm probability and enhances the HBR magnitude accordingly.

The concept that a change in harm probability — determined, in the current experiments, by different body postures relative to gravity — causes a change in DPPS shape is in line with previous findings, and provides an overarching narrative. For example, when the hit probability of objects in front of the body increases because of locomotion [9] or looming stimuli 1, 10, the peripersonal space expands forward. Altogether, these observations support the idea that the brain continuously calculates the probability of environmental threats hitting different body territories by integrating multimodal information into internal models of the physical laws of nature. This allows for successful estimation of the potential for harm of environmental events, and an appropriate adjustment of defensive responses.

## Author Contributions

R.J.B. and G.D.I. designed the study, analysed and interpreted the data, and wrote the paper. R.J.B. collected the data.

## Supplemental Information

Document S1. Experimental procedures, one figure, and one table
