# Supplementary material for: Gravitational cues modulate the shape of defensive peripersonal space
Source: Curr Biol. 2016 Nov 7;26(21):R1133–4. doi: 10.1016/j.cub.2016.09.025 (PMC5106387; doi:10.1016/j.cub.2016.09.025)
Supplement: Document S1. Experimental procedures, one figure, and one table [file mmc1.pdf]

Supplemental Information: Gravitational cues modulate the shape of defensive peripersonal space

Rory John Bufacchi and Gian Domenico Iannetti

Supplemental Data Items

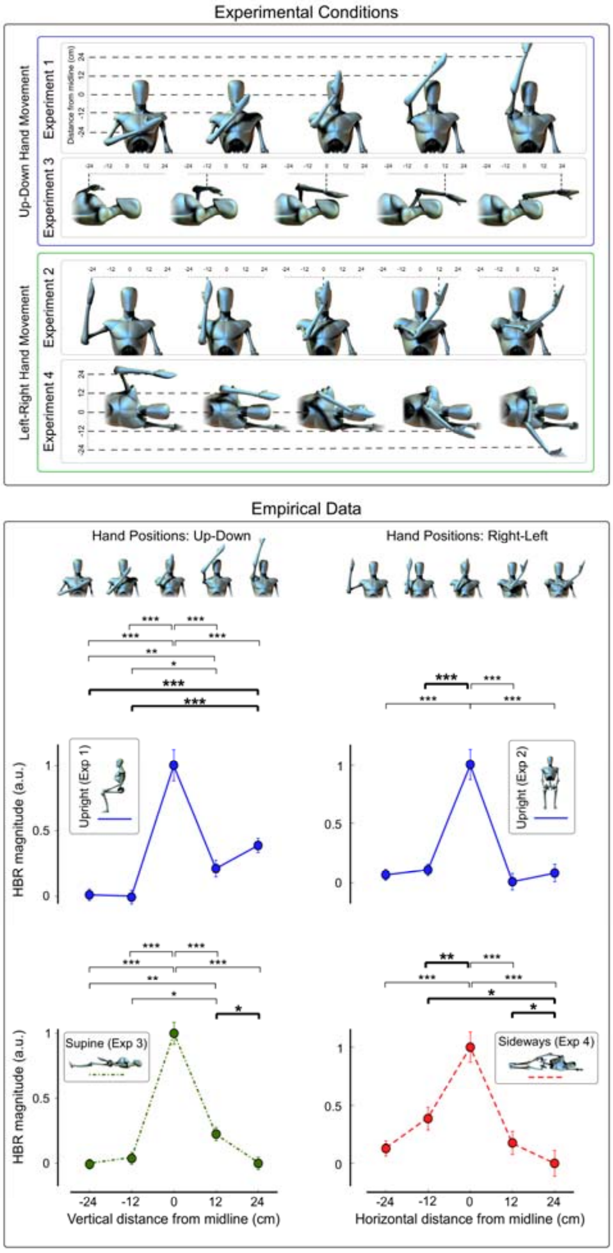

Figure S1 (Refers to Figure 1 in main text). *Top panel*. Hand positions at which the HBR was recorded. In all Experiments stimuli were delivered on a coronal plane located 4 cm from the nose. In Experiments 1 and 2, participants were upright. In Experiment 1, the stimulated hand was placed in 5 positions, along the body midline, symmetrically with respect to eye-level. In Experiment 2 these positions were along a horizontal line at eye-level, symmetrically with respect to the midline. In Experiment 3, participants were lying supine, and the stimulated hand was placed in the same positions as in Experiment 1, in head-centred coordinates. In Experiment 4, participants were lying on their side, and the stimulated hand was placed in the same positions as in Experiment 2, in head-centred coordinates. Once the participants had placed their hand in the correct position, the experimenter held the participants' forearm in place, so that the arm was relaxed completely. *Bottom panel*. Within-experiment post-hoc comparisons of HBR magnitude following hand stimulation in different positions (shown in figurines). Differences in statistical significance across body postures are highlighted in bold. Error bars indicate the standard error of the mean (SEM). Asterisks indicate: *$p<0.05$, **$p<0.01$, ***$p<0.001$.

Table S1 (Refers to figure 1 in main text)*. ANOVA post-hoc t-tests between all hand positions.*

Experiments 1 & 3		Upright (Exp 1)					Supine (Exp 3)				
		-24cm	*-12cm*	*0cm*	*12cm*	*24cm*	*-24cm*	*-12cm*	*0cm*	*12cm*	*24cm*
Upright (Exp 1)	*-24cm*		0.844	**0.000**	**0.005**	**0.000**	0.893	0.349	**0.000**	**0.001**	0.575
	-12cm			**0.000**	**0.012**	**0.001**	0.973	0.405	**0.000**	**0.000**	0.610
	0cm				**0.000**	**0.000**	**0.000**	**0.000**	0.893	**0.000**	**0.000**
	12cm					0.240	**0.001**	**0.023**	**0.000**	0.803	**0.003**
	24cm						0.000	**0.001**	**0.000**	0.322	**0.001**
Supine (Exp 3)	*-24cm*							0.344	**0.000**	**0.001**	0.550
	-12cm								**0.000**	**0.015**	0.679
	0cm									**0.000**	**0.000**
	12cm										**0.001**
	24cm										
Experiments 2 & 4		Upright (Exp 2)					On Side (Exp 4)				
		-24cm	*-12cm*	*0cm*	*12cm*	*24cm*	*-24cm*	*-12cm*	*0cm*	*12cm*	*24cm*
Upright (Exp 2)	*-24cm*		0.584	**0.000**	0.893	0.654	0.083	**0.005**	**0.000**	**0.025**	0.786
	-12cm			**0.000**	0.823	0.430	0.352	**0.008**	**0.000**	0.112	0.579
	0cm				**0.000**	**0.000**	**0.000**	**0.001**	0.899	**0.000**	**0.000**
	12cm					0.601	0.468	**0.025**	**0.000**	0.071	0.585
	24cm						0.056	**0.006**	**0.000**	**0.011**	0.994
On Side (Exp 4)	*-24cm*							0.061	**0.000**	0.256	0.194
	-12cm								**0.005**	0.321	**0.029**
	0cm									**0.001**	**0.000**
	12cm										**0.046**
	24cm										

P-values <0.05 are in bold.

Supplemental Experimental Procedures

Participants

We collected data from 29 right-handed healthy participants (19 women, 19-42 years, 24.2 ±5.0 years), who were all HBR responders [S1]. To obtain these HBR responders, we screened 55 subjects. Hence, 53% of subjects were HBR responders. This percentage is slightly lower than previous reports[S1–S3].

We conducted four experiments on 21 participants each. All participants who took part in Experiment 1 also took part in Experiment 3. Similarly, all participants who took part in Experiment 2 also took part in Experiment 4. This allowed us to perform a within-subject analysis. Participants gave written informed consent before taking part in the study. All procedures were approved by the local ethics committee.

Stimulation and Recording

Details of stimulation and recording procedures of Experiments 1 and 2 are reported elsewhere [S3]. Procedures of Experiments 3 and 4 were identical, except for the posture of the subjects. Briefly, transcutaneous electrical stimuli were delivered to the right median nerve at the wrist. Stimulus intensity was adjusted, in each participant, to elicit a reproducible blink reflex (mean = 37.5 ±15.4 mA). Stimulus duration was 200 µs, and the interval between two successive stimuli was 30 s. Electromyographic (EMG) activity was recorded from the *orbicularis oculi* muscle*,* bilaterally, using pairs of surface electrodes, with the active electrode over the mid-lower eyelid and the reference electrode lateral to the outer canthus. Signals were amplified and digitized at a sampling rate of 8,192 Hz (ISA 1004, Micromed, Treviso, Italy), and stored for offline analysis.

Experimental Procedures

Participants were seated in a comfortable chair. In each participant, we first determined the stimulus intensity able to elicit a well-defined and stable blink reflex in response to electrical stimulation of the median nerve at the wrist (HBR). This was achieved by increasing the stimulus intensity until a clear HBR was observed in three consecutive trials, or the participant refused a further increase of stimulus intensity [S2]. Only participants showing a reproducible HBR underwent further testing.

All experiments consisted of two blocks. In each block we delivered 5 stimuli at each of 5 hand position, for a total of 25 stimuli. In Experiments 1 and 3 the five positions were symmetrical with respect to eye-level, as follows (negative values denote positions below eye-level): ‘far-low’: -24 cm; ‘low’: -12 cm; ‘middle’: 0 cm; ‘high’: +12 cm; ‘far-high’: +24 cm. In Experiments 2 and 4 positions were symmetrical with respect to the vertical body midline, as follows (negative values denote positions on the participant’s right side): ‘far-right’: -24 cm; ‘right’: -12 cm; ‘middle’: 0 cm; ‘left’: +12 cm; ‘far-left’: +24 cm. These positions were marked on a board placed in front of the participant. Given that in preliminary experiments we observed that the effort of keeping the hand in the stimulated position had an effect on HBR magnitude, the participant’s arm was held in place by the experimenter, while the participant was instructed to relax their arm muscles. The order of hand positions was pseudo-randomized, with the constraint that no more than two consecutive stimuli were delivered for the same hand position.

Data analyses and statistics

EMG signals from each participant were high-pass filtered (55 Hz) and full-wave rectified. The HBR magnitude was calculated as the area-under-curve (AUC) of each single-trial response, separately for each recording site. We first averaged AUCs across ipsilateral and contralateral recording sites (as in [S3]) and then across the 10 trials at each hand position. Finally, we normalized the AUCs for each subject as Z-scores and for each experiment between 0 and 1.

To investigate the effects of hand position and body position we performed two different two-way repeated measures ANOVAs, one using the data pooled from Experiments 1 and 3, and the other using the data pooled from Experiments 2 and 4. In Experiments 1 and 3, the two experimental factors were ‘hand-position’ (five levels: ‘far-down’, ‘down’, ‘mid’, ‘up’ and ‘far-up’), and ‘body-position’ (two levels: ‘upright’ and ‘supine’). In Experiments 3 and 4, the two experimental factors were ‘hand-position’ (five levels: ‘far-left’, ‘left’, ‘mid’, ‘right’, and ‘far-right’), and ‘body-position’ (two levels: ‘upright’ and ‘sideways’).

Model fitting

In the previously validated geometric model of the DPPS [S3], the HBR magnitude is calculated from the geometric probability of the face being hit by a threat. The hitting probability in turn depends on the probability distribution of the directions in which the threat might be acting. Importantly for the objective of the current study, the previously observed rostro-caudal asymmetry of the DPPS (with larger extension above than below eye level) was modeled with a ‘gravity parameter’ (C_{grav}), consisting in a vector whose direction was fixed and matched the direction of gravity [S3]. Given that C_{grav} magnitude alters the probability distribution of hitting actions along the direction of the vector, a high C_{grav} value indicated a DPPS more extended above eye level.

In the three alternative versions of the model tested here, the direction of the C_{grav} vector was altered. In the ‘helmet’ model version, C_{grav} acted downward in *head-centred* coordinates, and it therefore did not reflect the influence of gravitational cues on the DPPS shape. In the ‘balloon’ model version, C_{grav} acted downward in *earth-centred* coordinates, and it therefore reflected the influence of gravitational cues on DPPS shape. Finally, in the ‘no gravity’ model,C_{grav} was set to zero and therefore the DPPS was symmetrical around the face, both horizontally and vertically.

The validity of each model version was assessed by its goodness of fit (GoF) to the mean HBR magnitudes at all hand positions. The GoF modelling approach compares the Chi-squared (χ^2) test statistic of the fit of any model version to the data, to a χ^2 distribution of the appropriate degrees of freedom, resulting in a GoF score and a corresponding *p* value[1]. Given that this approach requires that (1) the data are normally distributed, and (2) the variance across hand positions is equal, HBR magnitudes were first normalised to the ‘mid’ hand position in each subject for this analysis only, and then tested for normal distribution (using the Anderson-Darling test) and equality of variance (using the Bartlett’s test), as previously done [S3]. The results of these analyses showed that HBR magnitudes were normally distributed (p=0.37; H_0=normal distribution) and had equal variance (p=0.98; H_0=equal variance).

[1] Note that if the GoF-score is *larger* than 1.850 (which corresponds to the threshold of p=0.05 in the χ^2 distribution considered), the probability of the model being correct is *smaller* than 0.05, and the model must be rejected. Hence, the smaller the GoF score and the larger the p value of a model, the more strongly it is accepted.

Supplemental References

S1. Miwa, H., Nohara, C., Hotta, M., Shimo, Y., and Amemiya, K. (1998). Somatosensory-evoked blink response: Investigation of the physiological mechanism. Brain 121, 281–291.

S2. Sambo, C.F., Liang, M., Cruccu, G., and Iannetti, G.D. (2012). Defensive peripersonal space: the blink reflex evoked by hand stimulation is increased when the hand is near the face. J. Neurophysiol. 107, 880–889.

S3. Bufacchi, R.J., Liang, M., Griffin, L.D., and Iannetti, G.D. (2016). A geometric model of defensive peripersonal space. J. Neurophysiol. 115, 218–225.
